# Gastrointestinal Bleeding Is an Independent Risk Factor for Poor Prognosis in GIST Patients

**DOI:** 10.1155/2017/7152406

**Published:** 2017-05-15

**Authors:** Qi Liu, Yuji Li, Ming Dong, Fanmin Kong, Qi Dong

**Affiliations:** ^1^Department of Gastrointestinal Surgery, The First Hospital of China Medical University, Shenyang, China; ^2^Department of General Surgery, The People's Hospital of China Medical University, Shenyang, China

## Abstract

A retrospective analysis of prognosis of GIST was used to assess the prognostic effects of hemorrhage of digestive tract induced by mucosal invasion of primary gastrointestinal stromal tumors and related mechanisms. The conclusion is that GISTs with gastrointestinal hemorrhage are more likely to recur, which indicates poor prognosis. Therefore, gastrointestinal hemorrhage may be used as a significant indicator to assess the prognosis of patients.

## 1. Introduction

Gastrointestinal stromal tumor (GIST) is the most common soft tissue sarcoma in the digestive tract, and the stomach and small intestine are the most common sites. Liver metastases or peritoneal dissemination is the most common clinical malignant manifestations, but lymph node metastases are rare. Clinically, about 69% of patients with GIST are symptomatic, and gastrointestinal bleeding is the most common clinical symptom (in 30%–40% of cases) [1, 2]. Many patients seek medical treatment due to gastrointestinal bleeding. There are also many cases of patients that suffer an uncontrollably massive hemorrhage of the gastrointestinal tract and require emergency surgery. Many studies have focused on the prognosis of GIST [3, 4].

Of the factors influencing the prognosis of patients with stromal tumors, high-risk factors for recurrence include tumor size > 5 cm, mitotic count > 5 counts per 50 high-power fields (5/50 HPF), tumor rupture, postoperative recurrence risk > 50% [5, 6], and the location of the tumor. The evaluation of malignancy differs between gastric and nongastric GIST even with equal tumor size and the same mitotic counts [7]. Studies [8, 9] have shown that GIST is caused by mutations of the protooncogene c-KIT (60%–80%) or the platelet derived growth factor receptor (PDGFRA) (10–20%), suggesting the use for tyrosine kinase inhibitors (TKI) like imatinib mesylate for treatment of patients with GIST. The use of imatinib mesylate significantly improved the prognosis of patients with GIST, but the severe side effects and the high cost of these drugs limit widespread use. Therefore, it is necessary to select appropriate indicators to allow targeted therapy.

GIST tumor cells are thought to originate from Cajal cells [10], which are special cells that exist among smooth muscle cells. Exophytic growth, in which the tumor tends to grow outward beyond the surface of cells from which it originates, is the most common growth pattern of GIST. Tumor exophytic rupture can easily lead to abdominal metastasis. The National Comprehensive Cancer Network (NCCN) treatment guidelines and the National Institutes of Health (NIH) Risk stratification classify tumor rupture as a risk factor for recurrence but ignore another form of “rupture,” gastrointestinal bleeding triggered by local mucosal ischemic necrosis due to the mucosal invasion, or extrusion by the tumor. However, there are only few studies of the influence of GIST induced gastrointestinal bleeding on prognosis. To address this problem, the aim of our study was to investigate the impact of gastrointestinal bleeding on the prognosis of GIST and its possible mechanisms.

## 2. Materials and Methods

### 2.1. Materials

The clinical data of 301 patients with gastrointestinal stromal tumors treated surgically from September 2007 to March 2016 in the First Hospital of China Medical University were retrospectively analyzed. The inclusion criteria were as follows: (1) patients with primary gastrointestinal stromal tumors; (2) tumor diameter larger than 2 cm; and (3) no other primary malignant tumors. Only 178 of the 301 patients met these inclusion criteria. Follow-up by telephone to the patient or family and data from our outpatient department were used to determine the condition and survival status of these patients. Overall, we were unable to determine the outcome for eight cases, but the remaining 170 cases were analyzed statistically; among the 170 cases, 134 patients' clinical data are complete.

### 2.2. Method

All GISTs were pathologically diagnosed. The statistical data included age, sex, time of onset, tumor location, tumor size, the stage according to the TNM Classification of Malignant Tumors (TNM stage), bleeding status, whether R0 resection was performed, mitotic count, and whether targeted therapy was performed after surgery. We determined whether the patients suffered gastrointestinal bleeding based on the following criteria: (1) endoscopic confirmation; (2) digital subtraction angiography (DSA) confirmation; (3) CT or E CT; (4) low HGB and haematemesis or being positive in OB test; or (5) the surgical record or pathological confirmation.

### 2.3. Statistical Analysis

Statistical analysis was performed using SPSS 13.0 software. The Pearson chi-square test was used to analyze the enumeration data and ranked data. The measurement data were analyzed by the independent sample *t*-test or the nonparametric rank sum test. Additionally, we selected the statistically significant factors and performed multivariate analysis using logistic regression. For prognostic analysis, we did a univariate analysis using Kaplan-Meier survival analysis to find the statistically significant data. This data was then subjected to multivariate analysis using the Cox regression model. We defined values of *P* < 0.05 as statistically significant.

## 3. Results

### 3.1. Patient Information

The details of the patients in the study are presented in Table 1. The dataset contained one hundred and seventy patients with 89 males and 81 females (ratio of male to female of about 1.1 : 1). There were 92 patients less than 60 years old and 78 patients at least 60 years old. The age distribution was from 25 to 82 years old with an average of 58.3 years old. Sixty-three of the patients had gastrointestinal bleeding that was due to mucosal rupture of the tumor. The other 107 patients showed no signs of gastrointestinal bleeding. There were 106 cases of the primary tumor located in a gastric site and 64 in a nongastric site. The tumor diameters ranged from 3 cm to 30 cm with an average value of 7.1 cm. There were 59 cases of patients with a mitotic count greater than 5/50 HPF and 38 patients were treated with adjuvant therapy after surgery.

### 3.2. Factors Associated with Gastrointestinal Hemorrhage

The factors associated with gastrointestinal hemorrhage are presented in Table 2. The single-factor chi-square test showed that the following factors were associated with gastrointestinal hemorrhage: tumor size (*P* < 0.001), tumor location (*P* < 0.001), tumor T stage (*P* < 0.001), tumor M stage (*P* = 0.006), whether R0 resection was performed (*P* < 0.001), and positive expression of CD34 (*P* = 0.036). We subjected these statistically significant factors to multivariate analysis using logistic regression. The analysis suggested that the primary tumor location was an independent risk factor for gastrointestinal hemorrhage (*P* = 0.006) and GIST was more likely to cause gastrointestinal hemorrhage in the small intestine than in the stomach.

**Figure 1:**
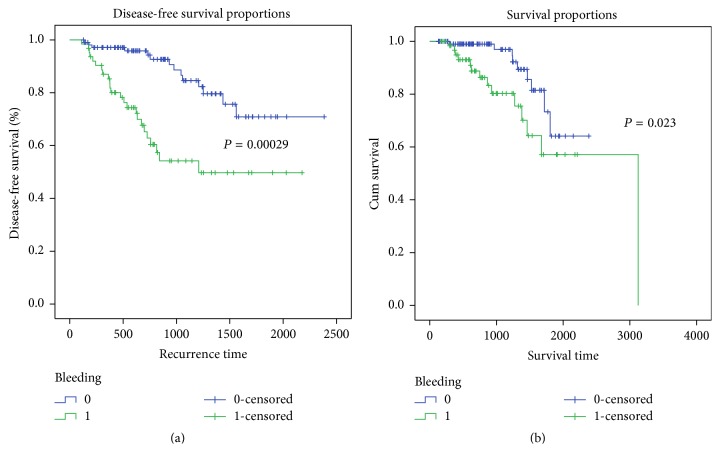
The Kaplan-Meier curve for GIST patients. (a) Recurrence time curve of the GIST patients with or without digestive tract bleeding. (b) Survival time curve for GIST patients with or without digestive tract bleeding.

Univariate and multivariate analysis for the prognostic factors of patients with GI bleeding are shown in Table 3. The Kaplan-Meier survival curve is shown in Figure 1. The data suggests that tumor recurrence is related to the following factors: tumor size (*P* < 0.001), tumor T stage (*P* < 0.001), tumor M stage (*P* = 0.013), presence of bleeding (*P* < 0.001), and whether R0 resection was performed (*P* = 0.004). We analyzed these factors described using the COX regression model. The results indicated that gastrointestinal bleeding was an independent risk factor for recurrence (95% CI: 1.105–4.919, RR: 2.332, *P* = 0.026).

The Kaplan-Meier survival curve (Figure 1) indicates that patient death is associated with tumor M staging (*P* = 0.011), bleeding (*P* = 0.023), R0 resection (*P* = 0.024), and positive expression of CD34 (*P* = 0.007). Analysis of these factors using the COX regression model showed that gastrointestinal hemorrhage is an independent risk factor for patient death (95% CI: 1.057–9.181, RR: 3.116, *P* = 0.039).

**Figure 2:**
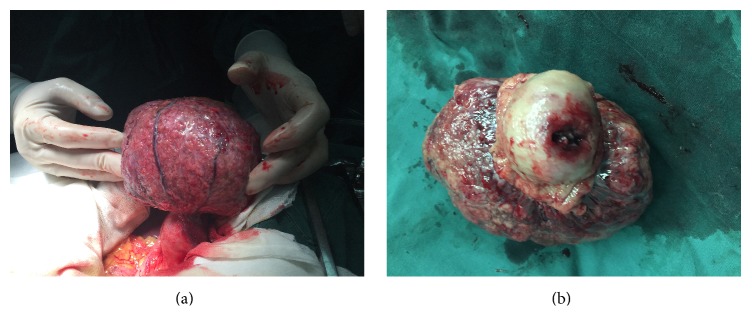
A typical case of gastric stromal tumor with gastrointestinal bleeding. (a) Gastric stromal tumor with a complete capsule. (b) Gastric stromal tumor with mucosal ulcer and digestive tract bleeding.

A typical case of a gastric stromal tumor with gastrointestinal bleeding is shown in Figure 2. A gastric stromal tumor with a complete capsule is shown for comparison with a gastric stromal tumor with mucosal ulceration and digestive tract bleeding.

## 4. Discussion

Mutations of KIT and PDGFRA are the most common causes of GIST [9, 11], which can develop in any part of the gastrointestinal tract with a different prognosis dependent on the tumor location. Tumor size, mitotic numbers, and tumor location are the most common risk factors for GIST risk stratification and are used as prognostic factors [7, 12, 13]. However, current prognosis methods for GIST have low accuracy and additional indicators are needed. Our results showed that clinical or pathological features such as the location and the size of the tumor are important factors affecting the prognosis of the patients, consistent with the previously known risk factors. We also found that digestive tract hemorrhage plays an important role in influencing prognosis. In addition, this study also found that patients with gastrointestinal bleeding may require R0 excision and are more likely to have a high tendency for metastasis than nonbleeding patients, which may explain why gastrointestinal bleeding impacts prognosis.

Gastrointestinal bleeding is a relatively familiar clinical manifestation with an incidence rate of about 30%–40% [1, 2]. In our study, patients with gastrointestinal bleeding accounted for about 37% of our patient set, consistent with the rate reported previous studies. The rupture of GIST will disseminate in the abdominal cavity, resulting in poor patient prognosis. Gastrointestinal bleeding caused by a tumor is one form of rupture. The rupture of digestive tract mucosa that is invaded by tumor can be considered as another form of tumor rupture, leading to the spread of tumor cells and thus affecting patient prognosis.

As we can see from Table 2, the T stage and M stage of the GIST were associated with gastrointestinal hemorrhage. To be more specific, GIST with higher T stage value was more likely to cause gastrointestinal hemorrhage. If there is a distant metastasis, the probability of gastrointestinal hemorrhage is dramatically increased, suggesting that gastrointestinal hemorrhage may be a significant factor inducing distant metastasis. In addition, the positive expression of CD34 (*P* = 0.064) has some significance. There was positive CD34 expression in 70%–80% of the GIST patients, consistent with use of the expression level of CD34 as an index to be applied early in the research and diagnosis of GIST. However, a potential relationship of CD34 expression to gastrointestinal hemorrhage requires additional study.

Gastrointestinal bleeding caused by GIST may affect prognosis because growth of the tumor can restrict the digestive tract mucosa, resulting in altered local mucosal blood supply. As a result, cell necrosis causes barrier damage and, together with digestive juices, this can ultimately cause ulcerative bleeding. Another kind of hemorrhage may be induced by blood vessel rupture when the tumor invades and erodes the mucosal or submucosal blood vessels [14]. In our study, the 63 cases of gastrointestinal bleeding included 28 cases of gastric bleeding and 35 cases of nongastric bleeding, with incidence rates of 26.4% and 54.7%, respectively. Comparing these incidence rates, we conclude that nongastric GIST was more likely to cause bleeding. Another study similarly reported that nongastric tumors are more inclined to evolve to necrosis [15]. Another possible explanation for these results is that the stomach is larger than the small intestine and other parts of the nongastric digestive tract, and the stomach is more resistant to extrusion by GIST.

GIST has a certain malignant tendency but only requires a wedge resection or a partial resection to remove the tumor completely without lymph node dissection, unlike other digestive system malignant tumors [16]. However, even if the tumor is completely resected, the likelihood of recurrence or metastasis after surgery is about 40%–50% [17]. Before 2001, surgery was the only treatment for GIST with a five-year survival rate of around 50% [18]. Recently, the use of TKI drugs like imatinib has improved patient prognosis and increased the probability of R0 resection [19]. However, it is worth pointing out that a GIST tumor is still likely to recur during the first 5 years after surgery, especially for patients in a high-risk group [20, 21]. Therefore, we should pay more attention to follow-up during this postsurgical period [4, 17, 22]. Although there are some related studies [23], there are no risk stratification criteria that consider gastrointestinal bleeding caused by GIST as a significant indicator. The significance of studying the prognosis of gastrointestinal hemorrhage caused by GIST suggests that increased attention is warranted for follow-up of patients with gastrointestinal bleeding caused by GIST. Patients with bleeding should be considered to have higher risk level, and doctors should be aware of the postoperative recurrence risk and reduce the threshold of postoperative targeted therapy for patients with gastrointestinal bleeding.

The Kaplan-Meier curves show that both the recurrence-free survival and the overall survival are shorter for patients with gastrointestinal bleeding caused by GIST. Gastrointestinal bleeding is an independent risk factor for GIST recurrence and death of the patients and should be considered a significant indicator of poor prognosis.

## Figures and Tables

**Table 1:** Clinical pathological characteristics of GIST patients.

Parameters	Numbers
Gender	
Male	89
Female	81
Age (years)	25–82
	(58.27 ± 10.54)
Tumor site (3–30 cm; AVE = 7.1 cm)	
Stomach	106
Intestine	64
Tumor size (cm)	
>5 cm	86
⩽5 cm	84
GI bleeding	
+	63
−	107
T stage	
T2	85
T3	59
T4	26
M stage	
Yes	5
No	165
Mitotic index	
>5/50 HPF	59
⩽5/50 HPF	77
R0 resection	
Yes	162
No	8
CD117	
+	139
−	5
CD34	
+	129
−	12
Dog1	
+	130
−	5
Adjuvant therapy	
+	38
−	80

**Table 2:** Factors associated with gastrointestinal hemorrhage.

Parameter	Stromal tumors with bleeding		*P*
	Yes	No	
Gender			
Male	36	53	0.346
Female	27	54	
Age (years)	58.71 ± 10.2	58.01 ± 10.8	0.874
Tumor site			
Stomach	28	78	<0.001
Intestine	35	29	
Tumor size (cm)			
≥5 cm	51	35	<0.001
<5 cm	12	72	
T stage			
T2	13	72	<0.001
T3	31	28	
T4	19	7	
M stage			
Yes	5	0	0.006
No	58	107	
Mitotic index			
≥5/50 HPF	23	36	0.590
<5/50 HPF	26	51	
R0 excision			
Yes	55	107	0.001
No	8	0	
CD117			
+	56	83	0.157
−	0	5	
CD34			
+	48	81	0.064
−	8	4	
Dog1			
+	49	81	0.651
−	1	4	

**Table 3:** Univariate and multivariate analysis for prognostic factors of patients with GI bleeding.

Parameters	Median recurrence (days)	Univariate analysis *P* (Kaplan-Meier)	Multivariate analysis hazard ratio (95% CI)	*P* (Cox-regression)
Gender (female/male)	1563/1815	0.883		
Age (>60/⩽60)	1797/1658	0.889		
Tumor site (gastric/nongastric)	1760/1706	0.914		
Tumor size (>5/⩽5)	1425/2076	<0.001	1.864 (0.525–6.622)	
T stage (T2/T3/T4)	2082/1503/1164	<0.001	1.277 (0.618–2.638)	
M stage (M0/M1)	1820/683	0.013	1.004 (0.103–9.763)	
Bleeding (positive/negative)	1366/1999	<0.001	2.332 (1.105–4.919)	0.026^*∗*^
R0 resection (yes/no)	1872/711	0.004	0.649 (0.083–5.043)	
Mitotic index per 50 HPF (<5/≥5)	1700/1650	0.154		
CD117 (positive/negative)	1685/1452	0.799		
CD34 (positive/negative)	1697/919	0.130		
DOG1 (positive/negative)	1660/760	0.090		
Targeted therapy (yes/no)	1649/1937	0.071		

^*∗*^ *P*-value is statistically significant; gastrointestinal hemorrhage is an independent risk factor.
